# Gallstone Pancreatitis: A Common but Often Overlooked Cause of Abdominal Pain in Post-Liver-Transplant Patients

**DOI:** 10.1155/2017/6047046

**Published:** 2017-10-22

**Authors:** Napatt Kanjanahattakij, Kamolyut Lapumnuaypol, Sanna Fatima, Eyob Feyssa

**Affiliations:** ^1^Department of Internal Medicine, Einstein Medical Center, Philadelphia, PA, USA; ^2^Department of Transplantation, Einstein Medical Center, Philadelphia, PA, USA

## Abstract

**Introduction:**

In general population, gallstone pancreatitis is the most common cause of pancreatitis. However, there are very few literatures that address this topic in post-liver-transplant patients.

**Clinical Case:**

A 69-year-old female who had a liver transplant in 2015 due to hepatocellular carcinoma and nonalcoholic steatohepatitis (NASH) cirrhosis. She had a recent episode of acute cellular rejection that was treated with high dose methylprednisolone 1 week prior to admission. She presented with severe epigastric abdominal pain associated with nausea and vomiting. Her laboratory studies showed significantly elevated serum lipase, AST, and ALT from her baseline. She underwent urgent Endoscopic Ultrasound (EUS) with Endoscopic Retrograde Cholangiopancreatography (ERCP) that showed common bile duct stone that was extracted.

**Discussion:**

Biliary sludge and stones accounted for 22% of late onset acute pancreatitis after liver transplant. Corticosteroids have been identified as one of the potential causes of drug-induced pancreatitis. However, she is more likely to have gall stone pancreatitis since she also had dilated common bile duct and intrahepatic duct. In addition, there was CBD stone noted on ERCP.

**Conclusion:**

Acute gallstone associated pancreatitis after liver transplant is not uncommon. Patients generally have good outcomes. Further prospective studies are warranted.

## 1. Introduction

Over the past decade, there has been an increase in the number of liver transplants in the United States. Over 70,000 adults are living with the liver allograft [1]. Biliary complication is one of the most common complications after liver transplant. The most common biliary complication after transplant is biliary stricture and bile leak [2]. Posttransplant bile ducts stone can also occur in 5–10% of liver-transplant recipients and it is associated with serious complication such as cholangitis and pancreatitis [3]. Acute pancreatitis can occur in 3–8% of post-liver-transplant patients [4]. However, there are very few case reports that specifically address this topic. We hereby report a case of a post-liver-transplant patient who presented to our center with abdominal pain and elevated serum lipase.

## 2. Case Presentation

We present a case of a 69-year-old female who had a liver transplant in 2015 due to hepatocellular carcinoma and nonalcoholic steatohepatitis (NASH) cirrhosis. Her posttransplant course was complicated by multiple episodes of acute cellular rejection. She had a recent episode of acute cellular rejection that was treated with high dose methylprednisolone 1 week prior to admission. Her immunosuppressive regimen included prednisone, mycophenolate mofetil, and tacrolimus.

She presented to the emergency department with severe epigastric abdominal pain associated with nausea and vomiting. She reported no recent alcohol or drug abuse. On abdominal examination, she had tenderness to palpation at epigastrium and right upper quadrant area without rigidity or rebound tenderness. Her laboratory studies showed significantly elevated serum lipase, AST, and ALT from her baseline (Table 1).

**Figure 1:**
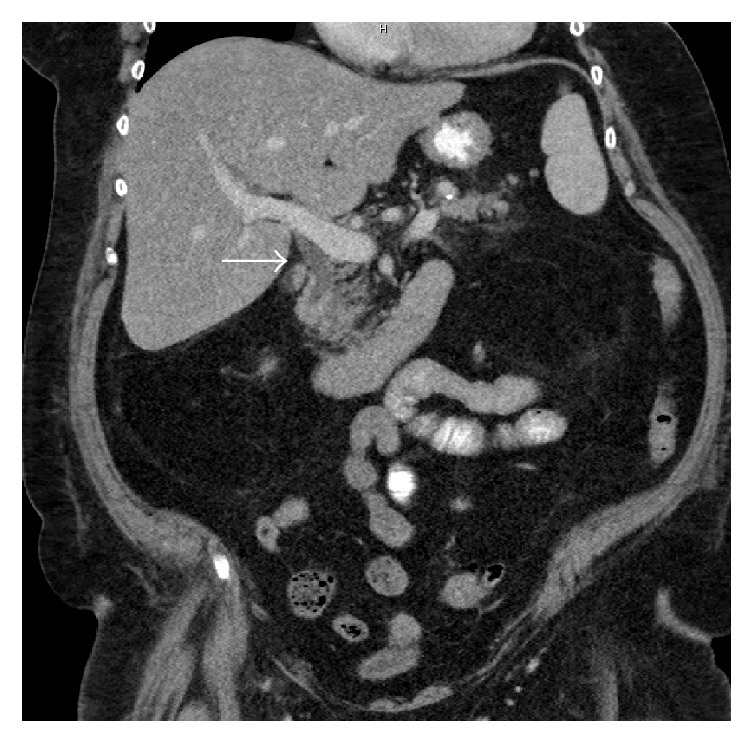
Dilated common bile duct (arrow).

Her abdominal Computed Tomography (CT) scans with contrast showed acute edematous interstitial pancreatitis with enlarged common bile duct and intrahepatic duct without any calculus or discrete obstruction (Figure 1).

Our differential diagnosis included gall stone pancreatitis and drug-induced pancreatitis secondary to high dose steroid. Her prednisone was stopped. She underwent urgent Endoscopic Ultrasound (EUS) with Endoscopic Retrograde Cholangiopancreatography (ERCP) that showed dilated common bile duct with 4 mm hyperechoic stone that was extracted. She was diagnosed with gall stone pancreatitis.

Her pain significantly improved after the stone extraction.

## 3. Discussion

Acute pancreatitis is one of the serious complications after liver transplant, which can occur in up to 8% of patients after liver transplant. Our patient presented with signs and symptoms of pancreatitis caused by CBD stone.

### 3.1. Diagnosis

The criteria for diagnosis of pancreatitis are similar in normal population and posttransplant patients. To establish the diagnosis, it requires two out of the three of the following criteria: (1) abdominal pain that is consistent with the disease, (2) serum amylase and/or lipase greater than 3 times of the upper normal limit, and (3) abnormal imaging that is consistent with the disease [5]. Isolated elevation of lipase or amylase is nondiagnostic without signs and symptoms of pancreatitis.

### 3.2. Etiology

Multiple studies reported various identified etiology of acute pancreatitis after liver transplant. In one study of 1832 patients undergoing liver transplant, postoperative acute pancreatitis occurred in 3% of the patients [6]. The etiology is believed to be from intraoperative manipulation and ischemia [4].

Infection has also been linked to post-liver-transplant acute pancreatitis. CMV and varicella-zoster viruses have been reported as rare causes of posttransplant acute pancreatitis [7–9].

Posttransplant ERCP is also common after liver transplant. The ERCP is commonly performed in posttransplant period to rule out biliary complications, such as biliary stricture that can occur in 5–15% after deceased donor liver transplant [2]. ERCP is usually required to correct these complications resulting in high incidence of post-ERCP pancreatitis up to 11% of post-liver-transplant pancreatitis [4, 10, 11].

Biliary sludge and stones accounted for 22% of late onset acute pancreatitis after liver transplant [11]. Corticosteroids are the first-line treatment for acute rejection in post-liver-transplant population. Corticosteroids have been identified as one of the potential causes of drug-induced pancreatitis [12].

Our patient received steroid for the treatment of her acute rejection. However, she is more likely to have gall stone pancreatitis since she also had dilated common bile duct and intrahepatic duct. In addition there was CBD stone noted on ERCP.

### 3.3. Biliary Stone after Liver Transplant

A large study of 1650 subjects showed that bile duct filling defects can occur in 5.7% of patients after liver transplant. 56% percent of bile duct filling defects are from biliary sludge or cast, and 32% are from stone [13].

In a case control study, patients with bile duct stone were more likely to have underlying bile duct pathology after transplant and higher triglyceride and cholesterol.

### 3.4. Management

Management of acute pancreatitis in post-liver-transplant patient is generally the same as in normal population. There is no specific guideline for this specific population. Management of pancreatitis involves intravenous hydration, bowel rest, and etiology specific management [4, 5].

Our patient underwent ERCP and the stone was removed and she had significant symptom improvement. ERCP in the period after liver transplant is generally a safe procedure. A retrospective study, which included 157 post-liver-transplant patients who underwent ERCP, showed that complication occurred in 15% of the patients. The most common complication of ERCP is pancreatitis followed by bleeding [14].

### 3.5. Prognosis

Late post-liver-transplant pancreatitis has similar prognosis with general population. Mortality rate could be up to 11% of the patients [4].

## 4. Conclusion

Acute gallstone associated pancreatitis in post-liver-transplant patient generally has good outcomes. There is a paucity of data regarding the understanding of disease and specific risk factors associated with it. Further prospective studies are warranted for better understanding of the disease.

## Figures and Tables

**Table 1:** Summary of laboratory results.

	Day 2	Day 1	Baseline
BUN (8–27 mg/dL)	32	38	33
Creatinine (0.6–1 mg/dL)	0.9	1.0	1.1
ALT (0–55 IU/L)	303	101	141
AST (5–34 IU/L)	246	42	33
Total bilirubin (0.2–1.2 mg/dL)	0.8	0.5	0.5
Direct bilirubin (0.1–1.5 mg/dL)	0.4	n/a	0.3
ALP (40–150 IU/L)	152	94	95
INR (0.9–1.1)	1.0	n/a	1.0
Lipase (8–78 IU/L)	530	186	n/a
Albumin (3.4–4.8 g/dL)	3.6	4.1	3.5

BUN = blood urea nitrogen, ALT = alanine aminotransferase, AST = aspartate aminotransferase, INR = international normalized ratio, and ALP = alkaline phosphatase.
